# Fraxin modulates lipid metabolism as well as gut flora to avert NAFLD

**DOI:** 10.3389/fphar.2025.1657966

**Published:** 2025-12-11

**Authors:** Yang Jing, Dong Huicong, Guo Xuanchi, Li Cao, Zhang Huanhuan, Guo Yongze

**Affiliations:** 1 School of Clinical Medicine, Hebei University of Engineering, Handan, Hebei, China; 2 Department of Gastroenterology, Affiliated Hospital of Hebei University of Engineering, Handan, Hebei, China

**Keywords:** systems biology, coumarins, lipid metabolism, non-alcoholic fatty liver disease, gastrointestinal microbiome, fatty acid transport protein 36

## Abstract

**Background:**

Public healthcare systems are heavily burdened by non-alcoholic fatty liver disease (NAFLD), which is the leading “chronic liver disorder” around the globe. Fraxin, a natural compound extracted from Fraxini cortex in traditional Chinese medicine, exerts hepatoprotective effects. However, the mechanism by which fraxin alleviates NAFLD remains elusive. This research looks into fraxin’s therapeutic potential in NAFLD management using an integrated experimental and pharmacological strategy.

**Methods:**

First, network pharmacology was used to identify core therapeutic targets of fraxin for NAFLD. Second, we built protein-protein interaction (PPI) networks, followed by “Gene Ontology (GO)” along with “Kyoto Encyclopedia of Genes and Genomes (KEGG)” pathways. Molecular docking validated the interaction of fraxin with its predicted targets. To confirm fraxin’s therapeutic effect *in vivo*, we built a “methionine-choline-deficient” (MCD) diet-induced NAFLD mouse model. Comprehensive assessments included liver function tests, hepatic triglyceride content, inflammatory marker measurement, mRNA expression for key lipid metabolism enzymes through reverse transcription-polymerase chain reaction, fatty acid translocase/cluster of differentiation 36 (FAT/CD36) expression through Western blotting, and 16S ribosomal RNA sequencing to assess changes in metabolic dysfunction and the gut microbiota.

**Results:**

Network pharmacology identified 34 potential fraxin targets in NAFLD. GO and KEGG analyses suggested that fraxin primarily treats NAFLD by modulating lipid metabolism and atherosclerosis-related signaling pathways. *In vivo*, fraxin significantly lowered liver index and visceral fat accumulation, reduced serum levels of “interleukin-6 (IL-6),” “aspartate aminotransferase,” “tumor necrosis factor-α (TNF-α)” and “alanine aminotransferase,” and decreased hepatic TG content. Furthermore, fraxin downregulated IL-6 and TNF-α expression and lowered the gene and protein levels of FAT/CD36, controlling key targets in signaling pathways related to lipid metabolism and atherosclerosis. Additionally, fraxin altered the gut microbial composition, reducing the Firmicutes/Bacteroidota ratio while increasing the abundance of Bacteroidota, Bacteroidia, Bacteroidales, Prevotellaceae, and Alloprevotella. Therefore, fraxin attenuated gut microbiota dysbiosis in mice caused by the MCD diet.

**Conclusion:**

Fraxin alleviates MCD diet-induced NAFLD by controlling lipid metabolism as well as restoring the homeostasis of gut microbiota.

## 1 Introduction

“Non-alcoholic fatty liver disease” (NAFLD), recoined as “metabolic dysfunction-associated fatty liver disease” (Eslam et al., 2020), is identified as a hepatic syndrome manifestation. NAFLD has been frequently linked to insulin resistance, hyperlipidemia, obesity, hypertension, type 2 diabetes mellitus, and gut microbiota dysbiosis (Svegliatiet al., 2020). It is characterized by ≥ 5% hepatic steatosis without any secondary causes like “chronic viral hepatitis” or “extreme alcohol drinking.” The NAFLD spectrum spans “simple fatty liver,” “cirrhosis,” “non-alcoholic steatohepatitis (NASH),” and finally “hepatocellular carcinoma.” NAFLD affects about 25% people worldwide, with China showing the highest rise in prevalence, reaching 29.6% over the past two decades (Zhou et al., 2020). Over 50% of people with NAFLD have overweight or obesity (≥24 kg/m^2^ body mass index, BMI). Approximately 40% do not have obesity (BMI <25 kg/m^2^); nevertheless, they still present with risk factors, namely, visceral obesity, high waist circumference, male sex, hypertension, and dyslipidemia. This phenotype is termed “lean NAFLD” (Ye et al., 2020). Untreated NAFLD rapidly progresses to NASH and cirrhosis. No drugs have yet received regulatory approval for NAFLD management. Lifestyle changes, such as dietary control and exercise, remain the key management strategies. However, poor adherence and difficulties in maintaining long-term changes increase the risk of disease progression. Therefore, investigating the molecular mechanisms underlying NAFLD and developing novel therapeutic targets are warranted (Guo et al., 2022). The “methionine–choline-deficient (MCD) diet” induces inflammation and hepatic steatosis, imitating the pathological characteristics of NAFLD. In the MCD model, 40% of energy is derived from sucrose and 10% from lipids, while lacking methionine and choline. It exhibits histological features similar to human NAFLD, serving as a valuable tool for drug screening and mechanistic research (Peng et al., 2020). Fraxin is a coumarin extracted from “Fraxini cortex.” This traditional Chinese medicine reduces inflammation and delays cell damage (Nam et al., 2021; Ferdous et al., 2024). Fraxin may alleviate acute liver injury by inhibiting the “nuclear factor kappa B (NF-κB) pathway” (Niu et al., 2017). However, its role in diet-induced NAFLD remains unclear, particularly in hepatocyte lipotoxicity and gut microbiota dysbiosis. Using network pharmacology and molecular docking techniques, this study explores the fundamental processes by which fraxin treats NAFLD. Per our knowledge, this novel study looks into fraxin’s efficacy in an “MCD-induced NAFLD mouse model.” Fraxin alleviates NAFLD by regulating “fatty acid translocase/cluster of differentiation 36 (FAT/CD36)” together with gut microbiota. These results not only provide a theoretical basis for the hepatoprotective actions of Fraxin but also elucidate its role as a natural therapeutic agent in NAFLD management.

## 2 Materials and methods

### 2.1 Experimental materials

“Beijing Vitonglihua Laboratory Animal Technology Co., Ltd.” (Certificate No.: 110011241105511485) provided 40 specific pathogen free C57BL/6Nifdc male mice (age: 42–48 days; body weight: 18–20 g). The Affiliated Hospital of Hebei University of Engineering’s central laboratory animal facility housed these animals. It had a light and dark cycle of 12 h each, 20 °C ± 2 °C temperature, and 55% ± 5% relative humidity, with unrestricted supply of water and food. The “methionine-choline sufficient (MCS) control” and “MCD” diets were provided by Jiangsu Meishen Biomedicine. The Beijing Herbal Fangyuan Pharmaceutical Group Co., Ltd. provided fraxin (aesculin) with ≥98% purity at a concentration of 20 mg per bottle (Lot: 20240601). The Nanjing Jiancheng Institute of Biological Engineering Co., Ltd. provided glutamate aminotransferase (GPT) (Lot numbers: 20240823), aspartate aminotransferase (GOT) (Lot number: 20240822), and triglyceride (TG) (Lot number: 20240914). Hangzhou Lianke Biotechnology Co., Ltd. provided “interleukin-6 (IL-6)” (Lot number: EK206/3-AW1) as well as “tumor necrosis factor-α (TNF-α)” (Lot number: EK282/4-AW1). Other reagents included reverse transcription kit (Lot number: 0000604660), quantitative polymerase chain reaction SuperMix (Lot number: 0000614550) (“Promega Inc.”, United States), total RNA extraction kit (Lot: 0000610379), bicinchoninic acid (BCA) protein quantification kit (“Beijing Solebo Co., Ltd.”; Lot number: 240010025), and FAT/CD36 antibody (“Abcam Inc.”, United States; Lot number: 1002656-13). Additionally, the following materials were used: Ultrapure water machine (Beijing Puxi General Instrument Company); precision balance (Matsu Trading Company); Sigma 3-30KS Centrifuge (Beijing Wuzhou Dongfang Technology Development Co., Ltd.), SCILOGEX shaker (Shanghai Yake Biotechnology Co., Ltd.), high-throughput cryogenic grinding equipment (Ningbo Xinyi Ultrasonic Equipment Co., LTD. Xinyi-24n), real-time fluorescence polymerase chain reaction (PCR) equipment (Thermo Fisher Scientific), −80 °C refrigerator (Qingdao Haier Company), iMark microplate reader (Berton Instruments, United States), developer (Cytiva), water bath with a magnetic stirrer (Fuwei Laboratory Instrument Factory), and a protein electrophoresis and membrane transfer series (Beijing Junyi Dongfang Electrophoresis Equipment Co., Ltd.).

### 2.2 Network pharmacology

Fraxin was selected as the target compound based on screening in the “Traditional Chinese Medicine Systems Pharmacology” (“https://tcmsp-e.com/”), per the following criteria: drug-likeness ≥0.18 as well as oral bioavailability ≥30%. Subsequently, its structural formula was extracted from “PubChem” (“https://pubchem.ncbi.nlm.nih.gov/”) and entered into “SwissTargetPrediction” (“http://www.swisstargetprediction.ch/”). “*Homo sapiens*” was the selected species while retaining interactions showing a prediction probability >0. To select disease-related genes, “nonalcoholic fatty liver” was imported into both “GeneCards” (“https://www.genecards.org/”) and “OMIM” (“https://www.omim.org/”). All genes were compiled, and duplicates were eliminated. A “Venn diagram” (“https://bioinfogp.cnb.csic.es/tools/venny/”) visualized the intersection between NAFLD-associated genes and fraxin targets; overlapping genes served as candidate targets. We used the “Search Tool for the Retrieval of Interacting Genes/Proteins” (STRING) database (“https://string-db.org/”) to identify “protein-protein interaction (PPI)” information. They were visualized in “Cytoscape 3.9.2,” where core targets were selected based on degree values within the network. “R software,” together with the Bioconductor packages “clusterProfiler,” “STRINGi,” and “Pathview,” facilitated “Gene Ontology (GO)” as well as “Kyoto Encyclopedia of Genes and Genomes (KEGG)” pathway enrichment analyses. The “MicroBioInfo” platform helped visualize the enriched terms. “GO” analysis comprises “biological process (BP),” “molecular function (MF),” and “cellular component (CC),” which annotate the role of candidate targets. KEGG analysis focuses on the key signaling pathways affected by fraxin in NAFLD management. Finally, the core targets and fraxin underwent molecular docking. The AlphaFold database yielded protein crystal structures, whereas PubChem comprised a small molecule library. Proteins underwent dehydration and hydrogen addition using “Autodock Tools 5.6.” “Autodock” and “Open Babel” programs enabled ligand processing. Docking was conducted using “Autodock,” with “PyMOL” visualizing binding conformations to foresee the binding affinity between fraxin and its core targets.

### 2.3 Animal experiments

#### 2.3.1 Establishment of NAFLD mouse model, intervention protocol, and sample collection

After 7 days of acclimation, 40 male mice (C57BL/6Nifdc inbred strain) were categorized (n = 10/group) at random: a blank control group (MCS) that was fed a standard diet; a model group (MCD) that was fed an MCD diet; and two intervention groups (MFL and MFH) that were fed the MCD diet along with and high (40 mg kg^-1^) as well as low (20 mg kg^-1^) fraxin doses administered intragastrically. Throughout the experiment, food intake, body weight, and behavioral characteristics were monitored. All mice were killed humanely after a 12-h fast after 4 weeks. To obtain serum, blood was drawn. Samples of fresh feces and liver were taken, quickly frozen in liquid nitrogen, and kept at −80 °C. The research protocol was licensed by the “Hebei University of Engineering Laboratory Animal Ethics Committee” (IACUC approval number: IACUC-Hebeu-2025-0005). Compliance with the “National Institutes of Health’s Guide for the Care and Use of Laboratory Animals” was maintained throughout all animal procedures.

#### 2.3.2 Histological analysis

For 24 h, liver tissues were preserved in 4% paraformaldehyde. They were dehydrated using a graded ethanol series, paraffin-embedded, and sectioned. Histological evaluation was conducted by visualizing lipid accumulation via “oil red O staining” as well as evaluating general morphology via “hematoxylin and eosin” (H&E) staining. A light microscope helped examine the sections, and pictures were captured for further examination. Finally, the hepatic steatosis degree was determined by calculating the hepatocyte percentage showing lipid droplets within the hepatic acinar structure. “ImageJ-win64 software” assisted in the semi-quantitative analysis of steatosis, determining NAFLD severity.

#### 2.3.3 Biochemical index analysis

As directed by the manufacturer, we measured “aspartate alanine aminotransferase (ALT)” as well as “aminotransferase (AST)” levels using biochemical assay kits (“Nanjing Jiancheng Bioengineering Institute,” Nanjing, China). A similar method measured the hepatic triglyceride (TG) proportion in liver homogenates. We measured TNF-α as well as IL-6 serum concentrations using “enzyme-linked immunosorbent assay kits” (Hangzhou Enzyme-linked Biotechnology Co., Ltd., China).

#### 2.3.4 Reverse transcription polymerase chain reaction

The “Eastep Super Total RNA Extraction Kit” homogenized about 20 mg of liver tissue to extract total RNA. “Sangon Biotech” (Shanghai, China) produced gene-specific primers for reverse transcription, and the “GoScript Reverse Transcription Kit” provided cDNA (Table 1). We used the “GoTaq PCR Master Mix” to conduct quantitative polymerase chain reaction. Real-time fluorescence data were gathered to ascertain gene expression. The “2^-ΔΔCt method” determined the relative gene expression: “fatty acid synthase (FAS),” “acyl-CoA oxidase (AOX),” “acetyl-CoA carboxylase 1 (ACC1),” “FAT/CD36,” “carnitine palmitoyltransferase 1α (CPT1α),” “liver-type fatty acid binding protein (LFABP),” “apolipoprotein B (ApoB),” “IL-6,” “microsomal triglyceride transporter (MTTP),” and “TNF-α.” mRNA levels in liver samples could be precisely measured because of this method.

**TABLE 1:** Primer sequence.

Gene	Forward primer	Reverse primer
FAS	TAT​CAA​GGA​GGC​CCA​TTT​TGC	TGT​TTC​CAC​TTC​TAA​ACC​ATG​CT
ACC1	ATG​GGC​GGA​ATG​GTC​TCT​TTC	TGG​GGA​CCT​TGT​CTT​CAT​CAT
CD36	GGA​GCC​ATC​TTT​GAG​CCT​TCA	GAA​CCA​AAC​TGA​GGA​ATG​GAT​CT
LFABP	ATG​AAC​TTC​TCC​GGC​AAG​TAC​C	CTG​ACA​CCC​CCT​TGA​TGT​CC
CPT-1	CTC​CGC​CTG​AGC​CAT​GAA​G	CAC​CAG​TGA​TGA​TGC​CAT​TCT
AOX	TAA​CTT​CCT​CAC​TCG​AAG​CCA	AGT​TCC​ATG​ACC​CAT​CTC​TGT​C
APOB	TTG​GCA​AAC​TGC​ATA​GCA​TCC	TCA​AAT​TGG​GAC​TCT​CCT​TTA​GC
MTTP	CTC​TTG​GCA​GTG​CTT​TTT​CTC​T	GAG​CTT​GTA​TAG​CCG​CTC​ATT
IL-6	CTC​CCA​ACA​GAC​CTG​TCT​ATA​C	CCA​TTG​CAC​AAC​TCT​TTT​CTC​A
TNF-α	ATG​TCT​CAG​CCT​CTT​CTC​ATT​C	GCT​TGT​CAC​TCG​AAT​TTT​GAG​A

#### 2.3.5 Western blotting analysis

To extract protein, 500 μL of Sheng’er Biotechnology’s radio-immunoprecipitation assay buffer (Shanghai) lysed liver tissue samples (approximately 20 mg each). A BCA protein quantification kit (“Solarbio,” Beijing) measured protein concentrations, and a microplate reader measured absorbance. We separated equal protein amounts using a 10% sodium dodecyl sulfate-polyacrylamide gel electrophoresis gel (“Yasen Biotechnology Co., Ltd.“, Shanghai) and put them onto “polyvinylidene fluoride membranes” (“Millipore,” Shanghai). We blocked membranes at room temperature in “Tris-buffered saline with Tween 20” (TBST) for 2 h with 5% skim milk and rinsed them with TBST three times (10 min per rinse). Primary antibodies against FAT/CD36 (“Abcam Inc.“, United States) (dilution: 1:1,000) were added to the membranes and incubated for the entire night at 4 °C. After TBST washing, we incubated membranes for 2 h in the dark at room temperature with “horseradish peroxidase-conjugated secondary antibodies” (dilution: 1:8,000). After another wash, an ultra-sensitive enhanced chemiluminescence reagent (“Sheng’er Biotechnology Co., Ltd.“, Shanghai) developed the membranes for 20 s in the dark. A system of automatic imaging visualized the bands. Finally, ImageJ software facilitated a densitometric analysis, and the internal reference for FAT/CD36 expression was glyceraldehyde-3-phosphate dehydrogenase (GAPDH). As a measure of relative protein expression, the semi-quantitative results are presented as the target protein to GAPDH intensity ratio.

#### 2.3.6 Gut microbiota analysis

Each mouse’s fresh fecal samples were stored in sterile Eppendorf tubes and immediately sent to “Wuhan Saierwei Biotechnology Co., Ltd.” (China) for sequencing the 16S rRNA gene with the “Illumina Novaseq 6,000” platform. Universal primers 308F and 806R amplified the 16S rRNA’s V3 to V4 region. Amplification products were barcoded during PCR, pooled, and sequenced in paired-end 250 bp mode. “Vsearch software” assembled the sequencing reads. Paired-end reads were merged based on an overlap of at least five bases. Additionally, chimeric sequences were eliminated. “Amplicon Sequence Variant” (ASV) clustering and abundance analysis were conducted using “QIIME2” (version 2022.2). Taxonomic annotation of ASVs was conducted using the “SILVA 138 database” within QIIME2.

#### 2.3.7 Online data acquisition and analyses

Single-cell RNA sequencing data of healthy liver tissues were acquired from the Human Protein Atlas database (https://www.proteinatlas.org/). For differential expression analysis, CD36 RNA-seq data from paired tumors and adjacent healthy liver tissues were selected using the “GEPIA2″ algorithm, which may be accessed at http://gepia2.cancer-pku.cn.

#### 2.3.8 Statistical analysis

“GraphPad Prism 8.0” and “SPSS 26.0” helped with all data analyses. The “mean ± standard deviation” (mean ± SD) value represents data that follows a normal distribution. The least significant difference (LSD) t-test helped perform pairwise comparisons after using one-way analysis of variance for comparing several groups. The definition of a statistically significant p-value was <0.05.

## 3 Results

### 3.1 Network pharmacology analysis

**FIGURE 1:**
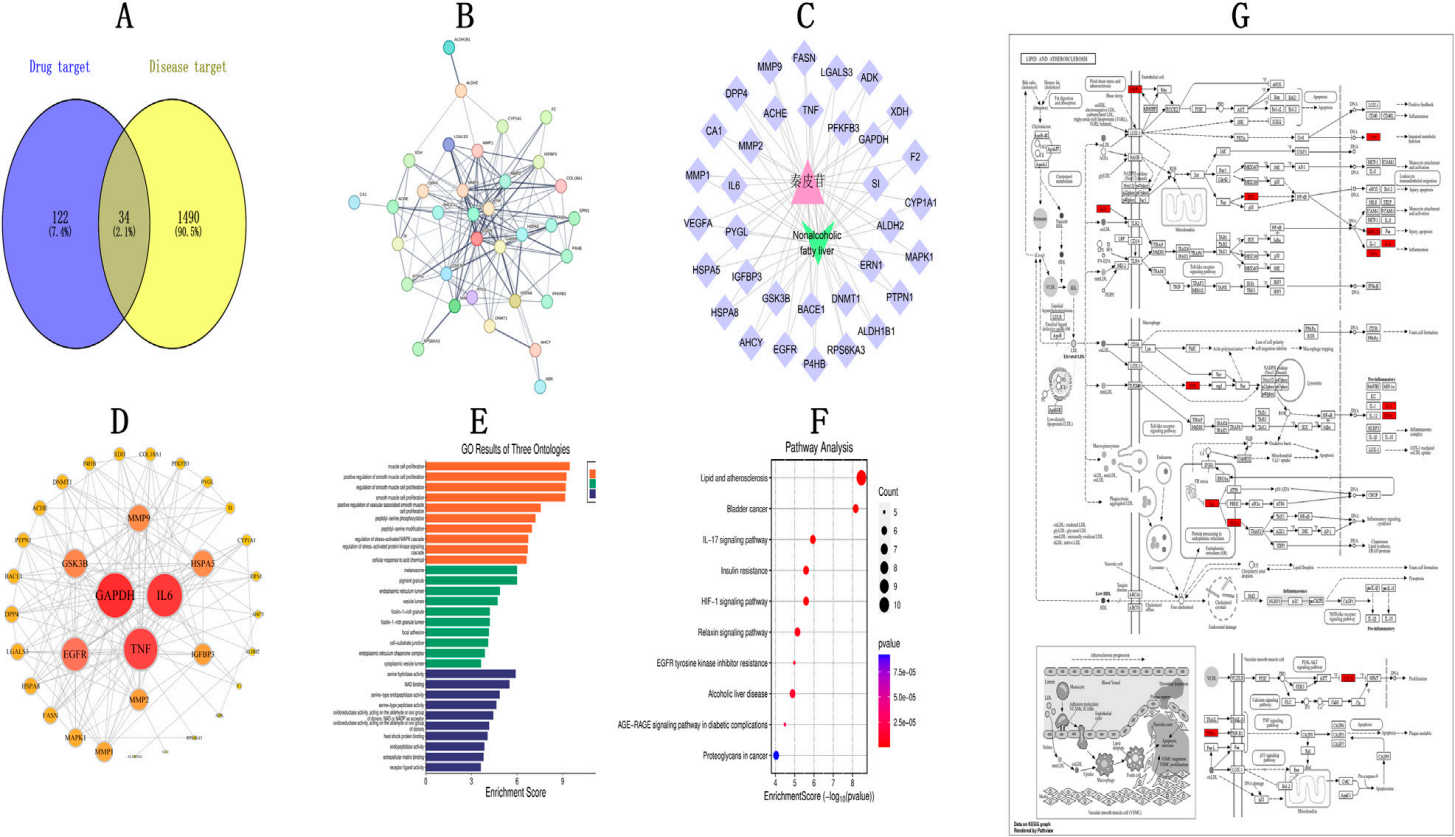
Network Pharmacology Analysis of Fraxin in NAFLD Management **(A)** Overlap between compound targets and NAFLD-linked targets. **(B)** PPI interaction network. **(C)** TCM-Target-Disease network diagram. **(D)** Protein-protein interactions. **(E)** GO analysis. **(F)** KEGG analysis (Y-axis: pathway names, X-axis: enrichment levels). **(G)** Lipid and atherosclerosis pathway. NAFLD, non-alcoholic fatty liver disease; TCM, Traditional Chinese Medicine; GO, Gene Ontology; PPI, protein-protein interaction; and KEGG, Kyoto Encyclopedia of Genes and Genomes.

In all, 1,490 gene targets linked to NAFLD, as well as 122 fraxin-related targets, were found. A Venn diagram showing the 34 overlapping targets that resulted from their intersection is shown in Figure 1A. To create a PPI network, these frequently used targets were fed into STRING (Figure 1B). Subsequently, Cytoscape 3.9.2 analyzed the network topology. The “Fraxin–target–disease network” was created by importing the type files and relevant nodes (Figure 1C). The PPI network comprised 34 protein nodes connected by 160 interaction edges (Figure 1D). Based on the degree values, the top 10 core targets were MMP1, IGFBP3, MMP2, MMP9, HSPA5, GSK3B, TNF, EGFR, IL6, and GAPDH, suggesting their potential in mediating fraxin’s impact in NAFLD management. To show the relevance of targets, we conducted “GO and KEGG pathway enrichment analyses.” Overall, 1,282 GO terms were enriched, containing 1,121 related to BP, 108 related to MF, and 53 related to CC. After applying a *P* < 0.05 threshold, the top 10 GO terms were selected to create a GO enrichment plot (Figure 1E). Enriched BPs included “smooth muscle cell and muscle cell proliferation,” “peptidyl-serine phosphorylation,” and the “stress-activated mitogen-activated protein kinase (MAPK)” cascade control. Enriched CCs were melanosomes, the endoplasmic reticulum (ER) lumen, vesicle lumen, fibrinogen-containing granule lumen, and ER chaperone complex. Enriched MFs included oxidoreductase activity (acting on aldehyde or ketone groups with NAD/NADP), heat shock protein binding, receptor ligand activity, and extracellular matrix binding. In KEGG pathway enrichment analysis, 211 pathways were enriched. After applying a *P* < 0.05 threshold, a bubble plot shows the top 10 pathways (Figure 1F). These pathways were predominantly related to lipid metabolism, atherosclerosis (Figure 1G), cancer-related pathways, the IL-17 signaling pathway, and insulin resistance, among others.

### 3.2 Molecular docking

**FIGURE 2:**
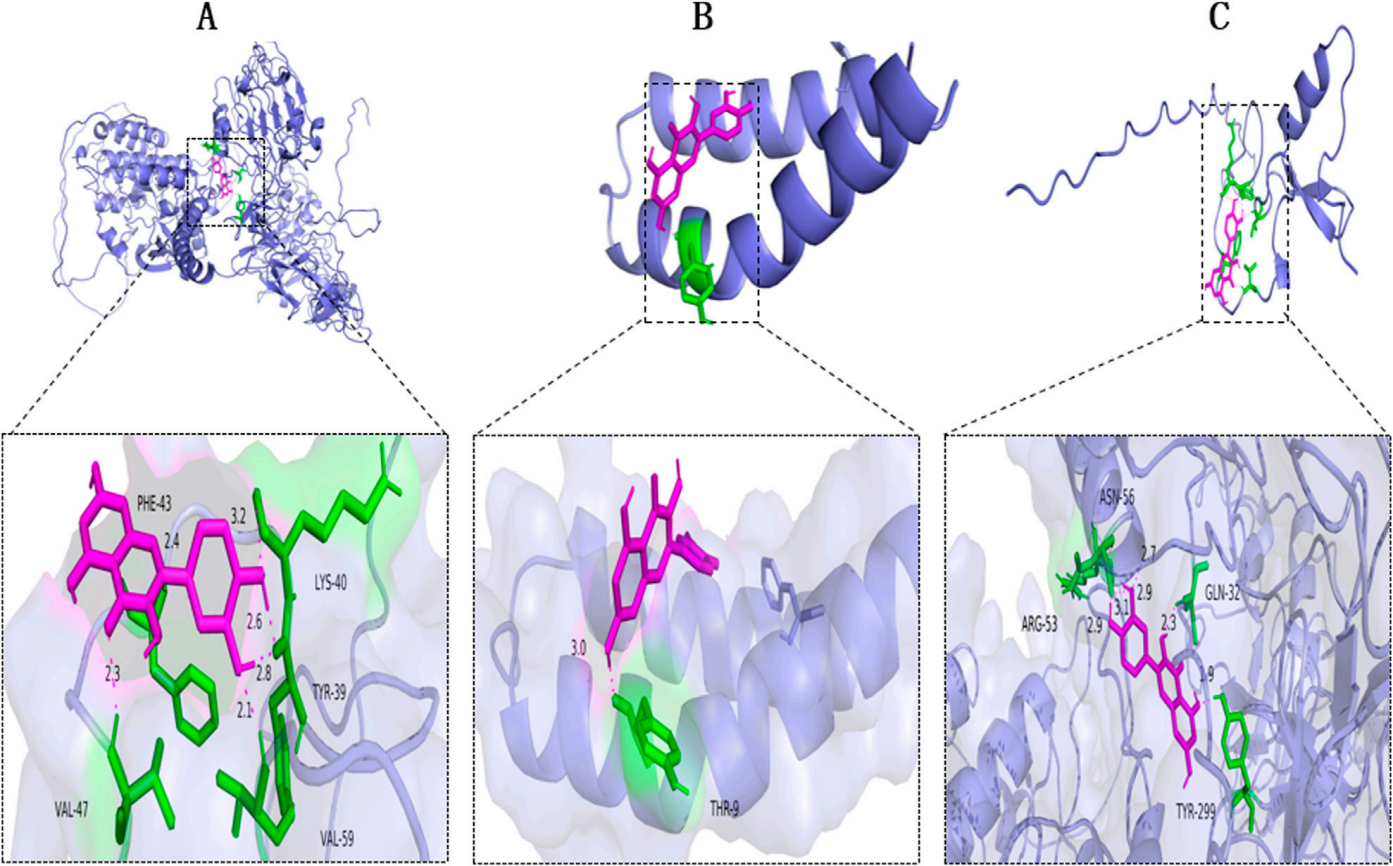
Molecular Docking of Fraxin with NAFLD-related Core Targets. Docking diagrams with **(A)** GAPDH, **(B)** IL-6, and **(C)** TNF-α. AFLD, non-alcoholic fatty liver disease; GAPDH, glyceraldehyde-3-phosphate dehydrogenase; IL-6, interleukin 6; and TNF-α, tumor necrosis factor-alpha.

To elucidate the interactions of fraxin with its target proteins, molecular docking analyses were conducted using GAPDH (PDB ID: P00533), IL-6 (PDB ID: AOA1C9J7T8), and TNF-α (PDB ID: HOYMI8), i.e., the top three core target proteins. Semi-flexible docking simulations predicted the binding affinities. A lower binding energy reflects a stronger binding affinity. The binding energies were −7.5 kcal/mol for Fraxin-GAPDH, −7.9 kcal/mol for Fraxin-IL-6, and –7.8 kcal/mol for Fraxin-TNF-α (Table 2). Fraxin demonstrated strong binding affinities toward IL-6 and TNF-α (Figure 2).

**TABLE 2:** PDB IDs of potential core targets for fraxin treatment and corresponding docking scores.

Targets	PDB DI	Binding energies/(kcal·mol - 1)
GAPDH	P00533	−7.5
IL-6	AOA1C9J7T8	−7.9
TNF-α	HOYMI8	−7.8

### 3.3 Effects of fraxin on liver index, body weight, liver biochemical indicators, and histopathology

**FIGURE 3:**
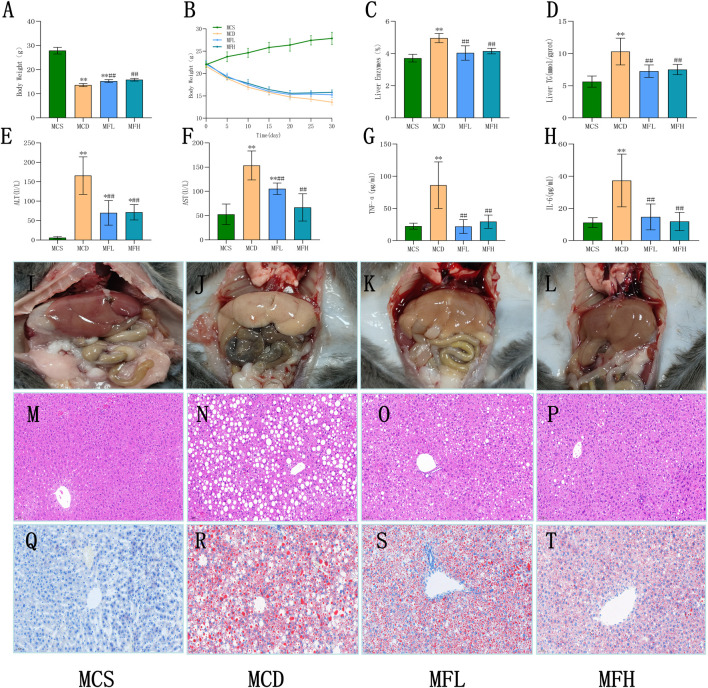
Fraxin improves biochemical indicators and histopathology in MCD diet-induced NAFLD mice **(A)** Body weight after 30 days. **(B)** Changes in body weight over 30 days. **(C)** Liver index after 30 days. **(D)** Liver TG content. **(E)** ALT. **(F)** AST. **(G)** TNF-α. **(H)** IL-6. **(I–L)** Gross appearance of mouse livers. **(M–P)** H&E staining at ×200 magnification. **(Q–T)** Oil Red O staining at ×200 magnification. **p < 0.01, *p < 0.05, compared with the MCS group; ##*p* < 0.01, #*p* < 0.05, compared with the MCD group. NAFLD, non-alcoholic fatty liver disease; IL-6, interleukin 6; TNF-α, tumor necrosis factor-alpha; H&E, hematoxylin and eosin stain; TG, triglyceride; MCD, methionine-choline-deficient; ALT, aspartate alanine aminotransferase; MCS, methionine-choline sufficient; and AST, aminotransferase.

Animals in none of the four groups experienced any mortality during the course of the experiment. High levels of physical activity and smooth, glossy fur were observed in the MCS group, suggesting that the mice were in good health. In contrast, focal hair loss, rough and disheveled fur, reduced activity, and progressive weight loss were observed in the MCD, MFH, and MFL groups. Toward the end of the study, the MCD group mice demonstrated lower body weights than the MCS group mice, indicative of malnutrition. Notably, mice in the MFL as well as MFH groups demonstrated higher body weights, implying the protective role of fraxin against diet-induced weight loss (Figures 3A,B). This hepatoprotective effect was also observed in the liver, as the liver index values reflected a decreased “liver weight relative to body weight” in the MFL and MFH groups (Figure 3C). Hepatic TG content and ALT, TNF-α, AST, and IL-6 levels were all significantly higher in the MCD group, suggesting lipid buildup (Figures 3D–H). These results were corroborated by gross morphological observations: livers from the MCS group were dark red, soft, and elastic, whereas those from the MCD group were pale, fragile, and showed diffuse lipid granule deposition on the surface. Moreover, they were susceptible to bleeding. A healthier liver appearance with less lipid deposition and increased tissue resilience was observed in the MFL and MFH groups (Figures 3I–L). These findings were supported by histological evaluations. Mice in the MCS group showed a normal hepatic architecture, with homogenous cytoplasm, polygonal hepatocytes, and centrally located nuclei. Contrarily, mice in the MCD group showed diffuse lipid vacuole accumulation, nuclear displacement, reduced cell contour clarity, and hepatocyte edema and deformation. These pathological changes were markedly alleviated in the MFL and MFH groups, with reduced vacuolization and partial hepatocyte restoration (Figures 3M–P). “Oil Red O staining” suggested that the MCS group showed minimal lipid buildup and an intact hepatic lobular structure. The MCD group showed focal droplet fusion along with extensive lipid droplet deposition, suggestive of severe steatosis. Fraxin administration markedly reduced both the area and intensity of staining, with diminished fusion of lipid droplets in the MFL and MFH groups (Figures 3Q–T). The MCD group had mild-to-moderate steatosis, per histological grading. Nevertheless, in the MFL and MFH groups, the degree of steatosis areas was either nonexistent or only marginally altered (Table 3).

**TABLE 3:** Degree of hepatic steatosis in mice by H&E staining.

Group	Number	Degree of steatosis			
		No steatosis	Mild steatosis (5%–33%)	Moderate steatosis (34%–66%)	Severe steatosis (>66%)
MCS	n = 6	6	0	0	0
MCD	n = 6	0	4	2	0
MFL	n = 6	1	5	0	0
MFH	n = 6	2	4	0	0

### 3.4 Gene and protein expression associated with lipid metabolism and atherosclerosis

**FIGURE 4:**
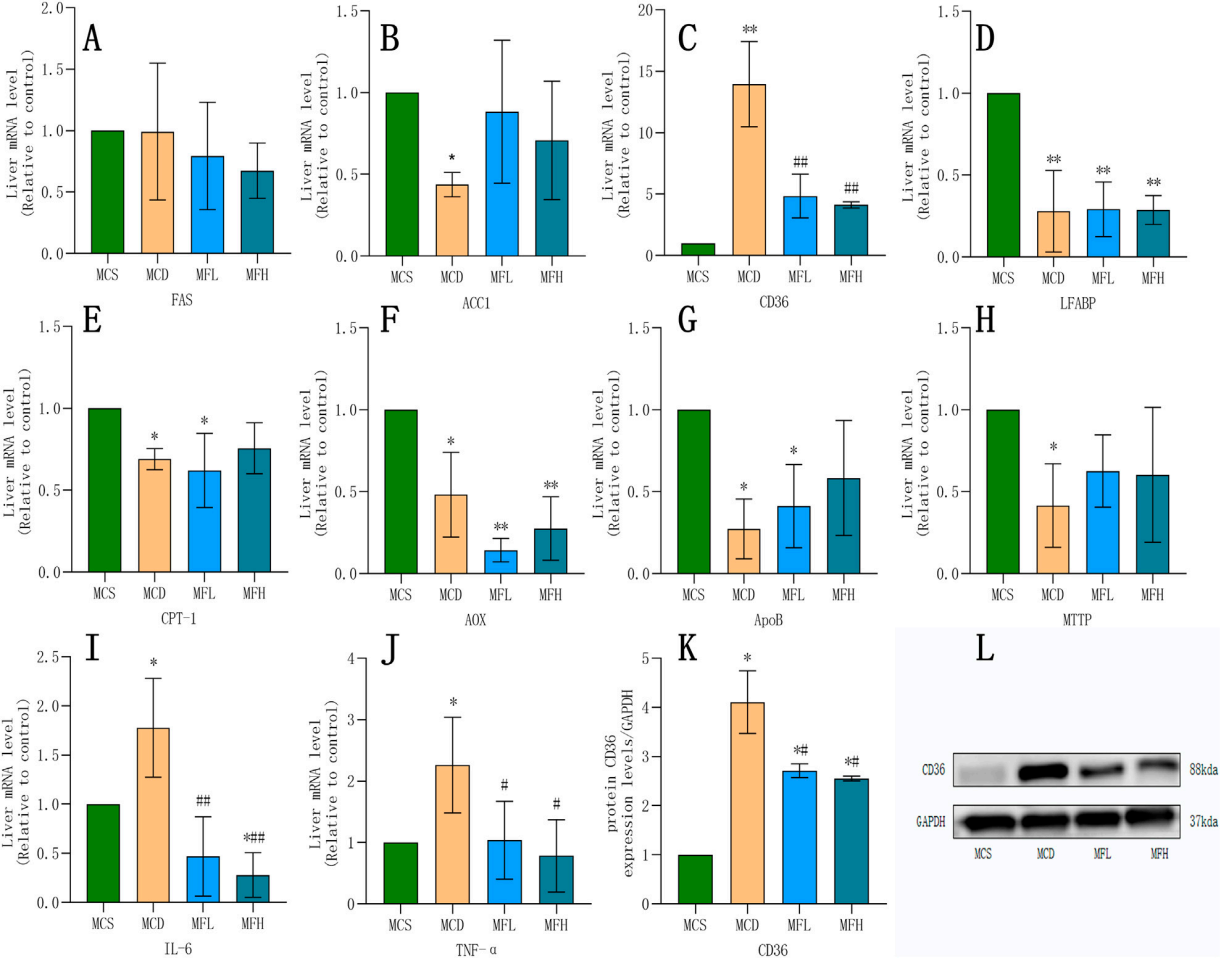
Impact of Fraxin on Gene and Protein Expression **(A–J)** mRNA expression of FAS, ACC1, FAT/CD36, IL-6, LFABP, CPT-1, AOX, ApoB, TNF-α, and MTTP across four groups. **(K,L)** Protein expression of FAT/CD36. **p < 0.01, *p < 0.05, compared with the MCS group; ##*p* < 0.01, #*p* < 0.05, compared with the MCD group. MCD, methionine-choline-deficient; MCS, methionine-choline sufficient; FAS, fatty acid synthase; ACC1, acetyl-CoA carboxylase 1; FAT/CD36, fatty acid translocase/cluster of differentiation 36; IL-6, interleukin 6; LFABP, liver-type fatty acid binding protein; CPT-1, carnitine palmitoyltransferase 1α; AOX, acyl-CoA oxidase; ApoB, apolipoprotein B; TNF-α, tumor necrosis factor-alpha; and MTTP, microsomal triglyceride transporter.

Relative gene expression of “FAS,” “ACC1,” “CPT1 α,” “AOX,” “FAT/CD36,” “LFABP,” “ApoB,” “MTTP,” “TNF-α,” and “IL-6” was compared across all four groups. The MCD group showed reduced FAS, ACC1, CPT1α, AOX, LFABP, ApoB, and MTTP expression, confirming a successfully developed NAFLD model. In contrast, FAT/CD36, TNF-α, and IL-6 expression were markedly raised in the MCD group. Both MFH and MFL groups showed significantly reduced “TNF-α,” “FAT/CD36,” and “IL-6” expression. According to an analysis of the Protein Atlas database, fibroblasts and Kupffer cells showed moderate FAT/CD36 expression, whereas healthy liver tissues showed low expression. Thus, FAT/CD36 is possibly involved in the development of liver inflammation and fibrosis. Furthermore, q-PCR and Western blotting helped examine mice livers from each group. The MCD group showed significantly higher FAT/CD36 expression than the MCS group. In contrast, the MFH and MFL groups showed significantly reduced FAT/CD36 expression (Figure 4).

### 3.5 Effects on gut microbiota

**FIGURE 5:**
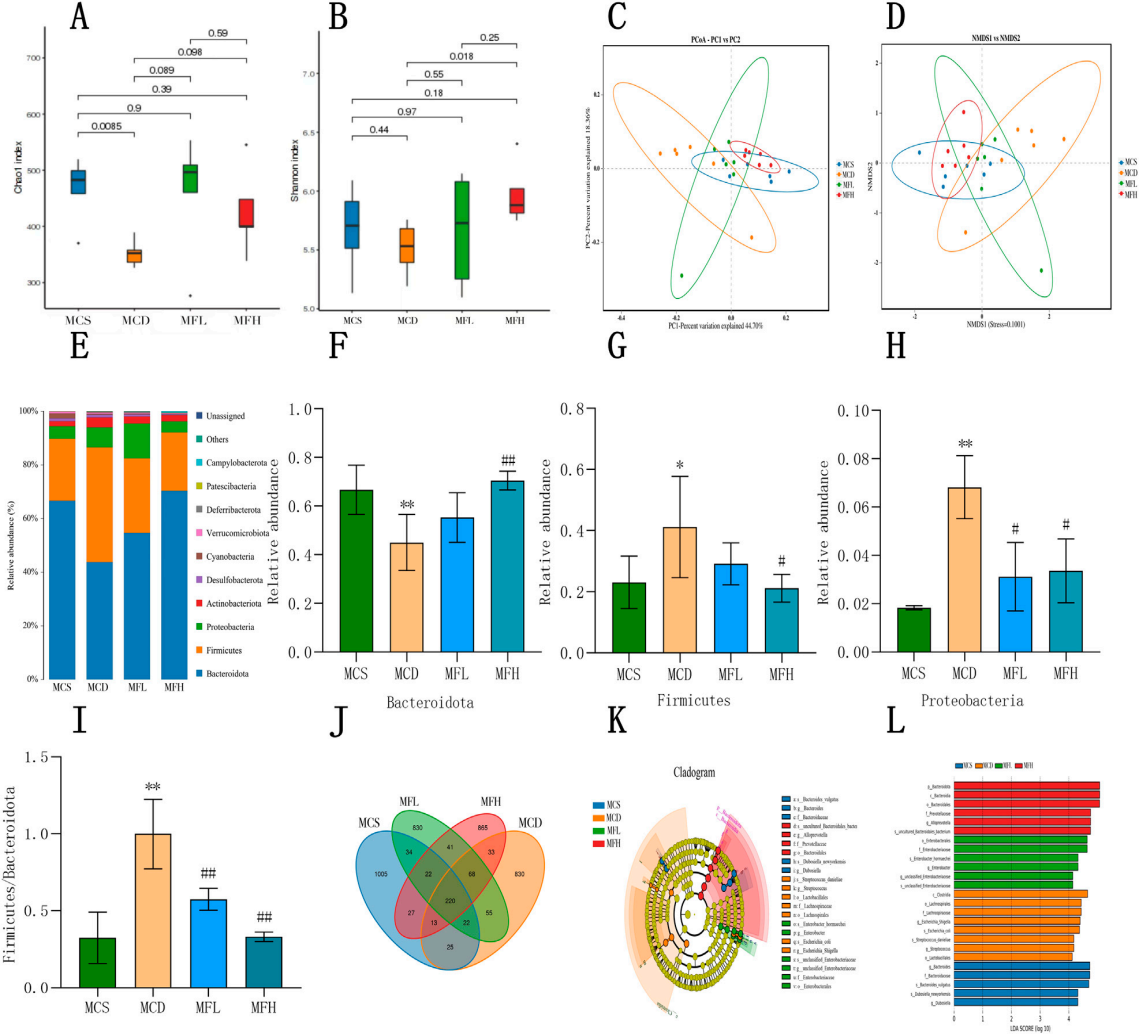
Fraxin’s effect on the Gut Flora **(A)** Alpha diversity analysis: Chao1 index before and after fraxin intervention in the MCD group. **(B)** Alpha diversity analysis: Shannon index. **(C)** PCA of gut flora. **(D)** NMDS of gut flora. **(E)** Relative abundance of gut flora at the phylum level. **(F–H)** Changes in the Bacteroidota, Firmicutes, and Proteobacteria abundance. **(I)** F/B ratio. **(J)** Distribution of gut flora. **(K,L)** Taxonomic cladograms and LDA score distribution histograms generated by the LDA effect size. **p < 0.01, *p < 0.05, compared with the MCS group; ##*p* < 0.01, #*p* < 0.05, compared with the MCD group. MCD, methionine-choline-deficient; MCS, methionine-choline sufficient; PCA, Principal Component Analysis; NMDA, N-methyl-D-aspartate; and LDA, Linear Discriminant Analysis.

Using 16S rRNA sequencing, the gut flora’s impact on fraxin’s therapeutic effects in NAFLD was investigated. Microbial composition was determined by clustering and annotating operational taxonomic units (OTUs) across the groups. The MCS, MCD, MFL, and MFH groups shared 220 OTUs. The MCD, MFL, and MFH groups had 830, 830, and 865 unique OTUs, respectively. Microbial dysbiosis in NAFLD is reflected in the decrease in OTU overlap with the MCS group (Figure 5J). Alpha diversity analysis suggested that both Chao1 as well as Shannon indices were lower in the MCD group, indicating reduced species richness and diversity. Despite statistical insignificance, both indices showed an increasing trend in MFL as well as MFH groups. Hence, fraxin may enhance the gut flora’s diversity and richness of in NAFLD, showing a trend toward microbial optimization (Figures 5A,B). Beta diversity analysis using “non-metric multidimensional scaling” as well as principal coordinate analysis demonstrated the differences among samples. Interestingly, we separated the samples between the MCS as well as MCD groups. However, the MFL and MFH groups indicated partial overlap with the MCS group, suggesting that fraxin modulates the gut microbiota structure in NAFLD (Figures 5C,D). Bacteroidota, Firmicutes, Proteobacteria, Actinobacteriota, Desulfobacterota, Cyanobacteria, Verrucomicrobiota, Deferribacterota, Patescibacteria, and Campylobacterota were the top 10 bacterial phyla. Notably, the dominant phyla remained consistent across the groups; nonetheless, their relative abundances differed. In the MCD group, Bacteroidota decreased, whereas Firmicutes and Proteobacteria increased. Conversely, the MFL and MFH groups exhibited reduced Firmicutes and increased Bacteroidota, suggesting gut microbiota restoration. Additionally, a higher “Firmicutes-to-Bacteroidota (F/B) ratio” was seen in the MCD group, which was significantly lower in the fraxin-treated groups (Figures 5E–I). Finally, linear discriminant analysis effect size analysis, together with an LDA threshold of 4, was conducted to identify the differential taxa. Compared with the MCS group, Clostridia, Lachnospirales, Lachnospiraceae, *Shigella* spp, and *Escherichia coli* were more abundant in the MCD group. Notably, the MFH group showed higher abundances of Bacteroidota and related taxa, indicating that high-dose fraxin significantly inhibited the microbial dysregulation induced by the MCD diet (Figures 5K,L).

## 4 Discussion

A chronic liver disorder that affects about 25% of people around the globe, NAFLD, is becoming a major problem for public healthcare systems. Notwithstanding its high prevalence, regulatory agencies have not yet authorized any pharmacotherapies for NAFLD. To mitigate lipid metabolism disorders, existing treatment modalities primarily include glycemic control, lipid-lowering medications, lifestyle modifications (exercise and dietary changes), and bariatric surgery (Rong et al., 2022). Intricate, multi-organ interactions involving countless biological pathways contribute to NAFLD development. Its pathogenesis has been explained by the “second strike hypothesis,” wherein hepatic lipid overload triggered by “insulin resistance,” a “high-fat diet,” “sedentary behavior,” and “obesity” is the first “strike,” and oxidative stress along with inflammation are the second “strike” (Buzzetti et al., 2016). A more complex “multiple hit hypothesis” takes into account immune dysregulation, gut microbiota imbalance, mitochondrial dysfunction, genetic predisposition, and impaired autophagy (Thanapirom and Tsochatzis, 2019). The disturbance of hepatic lipid homeostasis is central to NAFLD pathophysiology. Furthermore, disease progression is fueled by aberrant accumulation, which is caused by dysregulated lipid uptake, synthesis, oxidation, and export (Ipsen et al., 2018). A transmembrane glycoprotein belonging to the “class B scavenger receptor family,” CD36, is one of the major modulators of hepatic lipid accumulation. It facilitates “long-chain fatty acid” (LCFA) and oxidized “low-density lipoprotein” (oxLDL) absorption (Hao et al., 2020). Hepatic CD36 upregulation significantly accelerates NAFLD progression (Rada et al., 2020). CD36 is a multi-ligand pattern recognition receptor that interacts with LCFAs, oxLDL, collagen, and thromboxane (TSP-1/2). Mechanistically, ligand binding triggers downstream signaling cascades that mediate pro-apoptotic and anti-angiogenic effects in vascular endothelial cells, such as ERK, JNK, and p38 MAPK phosphorylation (Liu and Yin, 2025). Upstream CD36 expression regulators include the mammalian target of rapamycin (mTOR), an unusual mitogen-activated protein kinase that controls lipid synthesis and cell growth via the mTORC1/2 complexes. In NAFLD mouse models, hyperactivation of the mTOR pathway upregulates hepatic CD36 expression, exacerbating lipid deposition (Wang et al., 2014). In contrast, “AMP-activated protein kinase (AMPK),” a key energy sensor responding to AMP/ATP ratio changes, promotes fatty acid oxidation while suppressing anabolic processes. The inhibition of CD36 palmitoylation not only activates the AMPK pathway but also suppresses JNK-mediated inflammation, thereby attenuating hepatic lipid accumulation (Wang et al., 2018; Zhao et al., 2018). Furthermore, the “PI3K/AKT/mTOR signaling axis” coordinates metabolism, survival, and cell growth, with AKT serving as an upstream activator of mTOR. This axis has been implicated in tumorigenesis and chemoresistance (Ersahin et al., 2015). In murine models, the MCD diet is frequently used to mimic the pathological characteristics of NAFLD. This diet raises “reactive oxygen species” and causes a deficiency in “S-adenosylmethionine (SAMe),” which finally triggers the release of “TNF-α” as well as “IL-6” through the “gut-liver axis.” These alterations lead to hepatocyte injury and NAFLD progression. Oxidative stress caused by MCD generates a vicious cycle of “oxidative damage-inflammation-metabolic dysregulation” that culminates in cellular injury and apoptosis, damages proteins, lipids, and DNA, and finally activates hepatic immune cells (Palladini et al., 2022; Park et al., 2022). Natural products have drawn research interest because of their potential to modulate NAFLD-associated lipid metabolism. A coumarin derivative termed fraxin (C_16_H_18_O_10_), isolated from Fraxinus, shows promising antimicrobial efficacy and a favorable safety profile. Fraxin effectively reduces carbon tetrachloride-induced hepatotoxicity by altering MAPK-NF-κB signaling and improves diabetic renal tubulointerstitial fibrosis by blocking the NF-κB pathway (Zeng et al., 2023). Additionally, by activating the AKT pathway, fraxin delays diabetic renal fibrosis (Niu et al., 2017), providing novel insights into NAFLD treatment. In this study, fraxin significantly reduced “IL-6” along with “TNF-α” levels in MFL as well as MFH groups (P < 0.01), with parallel reductions in hepatic IL-6 along with TNF-α gene expression (P < 0.01). Fraxin alleviates hepatic inflammatory injury in NAFLD. The MCD group exhibited reduced gene expression of FAS, ACC1, CPT1α, AOX, LFABP, ApoB, and MTTP, compared with the MCS group, confirming successful model induction. However, both gene and protein expression of FAT/CD36 were raised in the MCD group (P < 0.01). Intriguingly, MFL as well as MFH groups showed significantly suppressed CD36 expression (P < 0.01). FAT/CD36 is central to lipid metabolism. To investigate its function in the liver, the expression and functional mechanisms underlying FAT/CD36 in human liver tissues and hepatocellular carcinoma were investigated. According to the results of single-cell RNA sequencing, FAT/CD36 is primarily expressed in fibroblasts and Kupffer cells in healthy liver tissues. TCGA database analysis showed that FAT/CD36 expression was significantly higher in hepatocellular carcinoma than in healthy liver tissues (Supplementary Figure S1). Therefore, by affecting liver inflammation and fibrosis, FAT/CD36 may facilitate the progression of liver cancer. We investigated how FAT/CD36 functions physiologically during the progression of liver diseases, which is in line with the traditional theory of liver disease development: “non-alcoholic fatty liver disease (NAFLD) – non-viral hepatitis – liver fibrosis – hepatocellular carcinoma.” qPCR and Western blot analyses confirmed that fraxin modulates FAT/CD36 expression at both the mRNA and protein levels. Therefore, we hypothesized that fraxin regulates FAT/CD36 expression in hepatic fibroblasts and Kupffer cells, thus influencing MCD diet-induced inflammatory and fibrotic responses. In the future, further mechanistic studies will be conducted. In summary, FAT/CD36 is strongly associated with the prognosis of NAFLD and hepatocellular carcinoma. In mice models, fraxin intervention significantly improved liver function. In healthy individuals, the gut flora controls host physiology, including pathogen resistance, immune modulation, and biosynthesis of essential metabolites, such as “bile acid derivatives” as well as “short-chain fatty acids” (Matenchuk et al., 2020). Bacterial metabolites and microbial components, such as lipopolysaccharide and peptidoglycan, can translocate through portal circulation when intestinal barrier integrity is compromised. By activating stellate cells as well as hepatic Kupffer cells, this microbial translocation may promote sterile inflammation, exacerbated oxidative stress, and hepatic fibrosis in NAFLD (Fang et al., 2022). The “multiple-hit” hypothesis of NAFLD is largely influenced by gut dysbiosis (Aragonès et al., 2019). Hepatic homeostasis and fibrotic progression are regulated by the gut–liver axis, which enables a two-way signal exchange between the gut flora and the liver (Ji et al., 2019; Fianchi et al., 2021). Notably, the gut flora can metabolize choline, a crucial methyl donor, to produce trimethylamine (TMA). Flavin-containing monooxygenases (FMOs) oxidize “TMA” to “trimethylamine-N-oxide” (TMAO). By reducing the hepatic bile acid pool and altering lipid along with glucose homeostasis, TMAO impairs the metabolism of fats and cholesterol. Therefore, the reduction of choline bioavailability caused by gut dysbiosis may increase susceptibility to NAFLD (Li et al., 2022). Despite similar dominant phyla (“Bacteroidia,” “Proteobacteria,” and “Firmicutes”) in both MCD and MCS groups, the former showed a significantly elevated F/B ratio, indicating an imbalance in the gut flora. A significant decrease in the F/B ratio (P < 0.01) was observed in both MFL and MFH groups (*P* < 0.01). Furthermore, the MCD group displayed higher relative abundances of potentially harmful bacteria linked to hepatic inflammation and intestinal barrier dysfunction, such as Clostridia, Lachnospirales, Lachnospiraceae, *Shigella* spp, and *E. coli*. In contrast, the MFH group exhibited increased relative abundances of beneficial bacteria, including Bacteroidota, Bacteroidia, Bacteroidales, Prevotellaceae, and Alloprevotella. These taxa maintain gut health, promote nutrient absorption, and inhibit harmful bacterial growth. By combining “molecular docking,” network pharmacology, and “experimental validation,” this study systematically elucidates the dual mechanism of fraxin in ameliorating NAFLD. Fraxin mitigates lipid accumulation by inhibiting FAT/CD36 and regulating intestinal flora. The use of an MCD diet-induced mouse model, which is considered for its histopathological similarity to human NAFLD, offers strong preclinical evidence for Fraxin’s therapeutic potential. Additionally, this novel study shows that fraxin can improve intestinal microbial balance by enriching beneficial taxa like Alloprevotella and lowering the F/B ratio. With regard to the use of natural compounds in NAFLD intervention, these results provide a conceptual framework.

In this study, we first created an MCD diet-induced NAFLD mouse model. Despite its widespread use, the MCD model does not accurately mimic the pathophysiological characteristics of human NAFLD. For example, it does not account for metabolic syndromes commonly observed in human NAFLD, such as insulin resistance and obesity. The MCD model can replicate most physiological characteristics of NAFLD, and weight loss induced by the MCD diet has been consistently observed in animal models. Upon analyzing this phenomenon, we concluded that weight loss in the MCD diet is primarily attributable to sarcopenic obesity, a pathological state in which sarcopenia and obesity coexist, which is caused by impaired protein synthesis and muscle loss resulting from methyl and choline deficiency rather than fat reduction. Fraxin enhances hepatic lipid metabolism and reduces inflammation; however, it cannot compensate for systemic methyl and choline deficiencies. Therefore, it does not reverse the weight loss caused by nutrient deficiency. However, fraxin may reduce the incidence and progression of NAFLD by mediating the suppression of FAT/CD36 expression and modulating the gut microbiota in tandem. Future studies should utilize other models (such as high-fat diet models) to validate these findings.

## 5 Conclusion

In conclusion, this study used network pharmacology and molecular docking technology to predict fraxin targets and pathways in NAFLD management. Additionally, animal experiments validated that fraxin alleviates hepatic lipid accumulation in NAFLD. Furthermore, it restores gut microbiota homeostasis by enriching beneficial gut microbiota. These findings systematically demonstrate that fraxin mitigates NAFLD through the “gut-liver axis” cycle, and its pharmacological mechanism may be associated with suppressed FAT/CD36 gene expression.

## Data Availability

The data presented in the study are deposited in the EBl Metagenomics repository, accession number PRJEB104885 (ERP186110).

## Supplementary material

SUPPLEMENTARY FIGURE S1 CD36 may play an important role in the disease progression from hepatitis to liver cirrhosis and hepatocellular carcinoma. **(A)** Single-cell RNA sequencing (scRNA-seq) revealed that CD36 is predominantly expressed in fibroblasts and Kupffer cells in normal liver tissue. **(B)** The expression level of CD36 was significantly higher in hepatocellular carcinoma (HCC) tissues compared to peritumoral liver tissues.
