# Supplementary figures and images for: Fraxin modulates lipid metabolism as well as gut flora to avert NAFLD
Source: Front Pharmacol. 2025 Dec 11;16:1657966. doi: 10.3389/fphar.2025.1657966 (PMC12738347; doi:10.3389/fphar.2025.1657966)

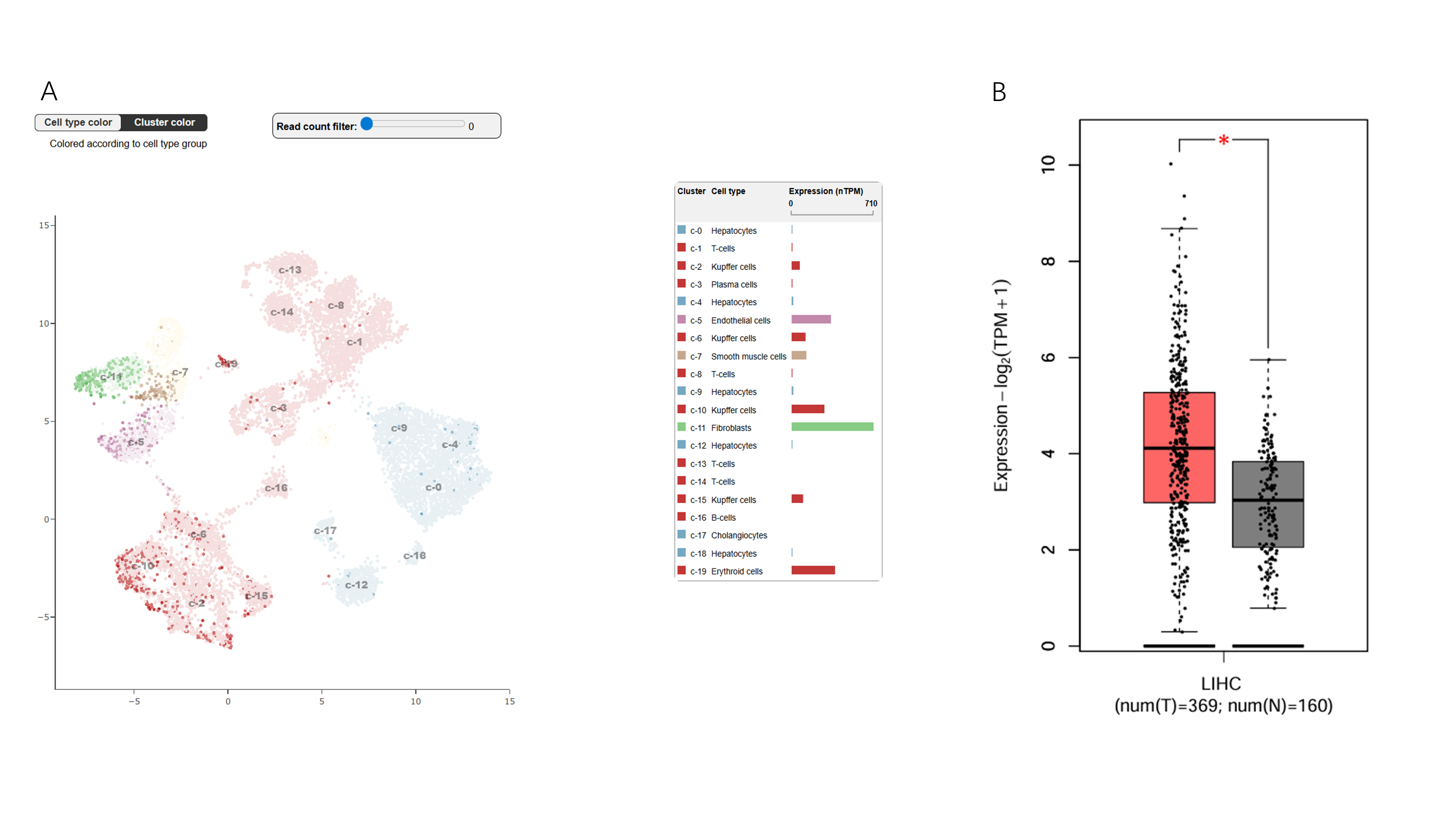

Supplement: Supplementary file 1 [file Image1.tif]
